# Inhibition of Matrix Metalloproteinase-7 Attenuates Subpleural Fibrosis in Rheumatoid Arthritis-Associated Interstitial Lung Disease

**DOI:** 10.3390/biomedicines13071581

**Published:** 2025-06-27

**Authors:** Li Xiong, Li-Mei Liang, Shu-Yi Ye, Xiao-Lin Cui, Shi-He Hu, Chen-Yue Lian, Wen-Jia Sun, Yang-Ping Lv, He-De Zhang, Meng Wang, Fei Xiang, Liang Xiong, Hong Ye, Wan-Li Ma, Lin-Jie Song

**Affiliations:** 1 Department of Respiratory and Critical Care Medicine, Union Hospital, Tongji Medical College, Huazhong University of Science and Technology, Wuhan 430022, China; 2 Key Laboratory of Respiratory Diseases of National Health Commission of China, Wuhan 430030, China; 3 Department of Pathophysiology, School of Basic Medicine, Tongji Medical College, Huazhong University of Science and Technology, Wuhan 430030, China

**Keywords:** rheumatic diseases, rheumatoid arthritis, RA-ILD, pleural fibrosis, collagen-I, MMP-7

## Abstract

**Background**: Rheumatoid arthritis-related interstitial lung disease (RA-ILD) is a significant complication of RA which lacks effective treatments with high mortality. This study aimed to investigate the role of matrix metalloproteinase-7 (MMP-7) in mediating RA-ILD. **Methods**: Based on the database of RA-ILD patients, a bioinformatics analysis was performed. A protein–protein interaction (PPI) network focusing on MMP-7 was simulated. Pleural mesothelial cells (PMCs) were treated with RA-ILD patients’ serum or RA-ILD-related inflammatory factors, and the protein expressions of collagen-I and MMP-7 were examined. An arthritis model was established using complete Freund’s adjuvant (CFA). Changes in the weight and joints of mice were recorded, and lung tissues were evaluated by Masson staining and Sirius red stain techniques. MMP-7 inhibitor, MMP-7 siRNA and MMP shRNA lentivirus were used to inhibit MMP-7 and investigate changes in collagen-I and fibrosis in vivo and in vitro. **Results**: MMP-7 was found to be significantly expressed in RA-ILD lung tissue by bioinformatics analysis, and MMP-7 to maybe interact with collagen-I. In vitro experiments indicated cytokines IL-1β, IL-6 and TNF-α promoted MMP-7 and collagen-I expression in PMCs. Serum obtained from patients with RA-ILD also upregulated MMP-7 and collagen-I expression in PMCs. Inhibition of MMP-7 with MMP-7 siRNA or MMP inhibitor prevented collagen-I synthesis in PMCs. In vivo, CFA induced arthritis and subpleural lung inflammation in rats, but the MMP-7 inhibitor and MMP-7 siRNA attenuated CFA-induced lung inflammation and subpleural lung fibrosis. **Conclusions**: MMP-7 mediated subpleural lung inflammation as well as fibrosis in RA-ILD. It provided theoretical and experimental support for MMP-7 being a therapeutic target in RA-ILD.

## 1. Introduction

Rheumatoid arthritis-related interstitial lung disease (RA-ILD) is characterized by joint manifestations [1] and diffuse interstitial pulmonary alterations [2]. The prognosis of RA-ILD is poor [3,4,5]. The median survival period of RA-ILD is 2.6~3.5 years [5,6]. The histological and pathological types in RA-ILD patients include non-specific interstitial pneumonitis (NSIP) and usual interstitial pneumonitis (UIP) [7,8,9]. UIP manifests as the progression of fibrotic diseases, with subpleural and paraseptal matrix deposition [10,11,12,13], while NSIP is characterized by inflammatory infiltration and thickening of the alveolar walls [11,13,14,15]. Unfortunately, UIP is the main histological type in RA-ILD. Treatments for RA-ILD are limited. Thus, the mechanism and new treatment strategy urgently need to be explored.

Matrix metalloproteinases (MMPs) are a general term for a class of proteases that can degrade extracellular matrixes and non-matrix proteins. MMPs play significant roles in various diseases such as tumors and fibrotic diseases. MMP-7 is among the smallest molecules within the MMP family. Based on molecular structures, collagen triple helix can be cleaved by the MMPs’ domains, indicating a direct interaction between MMP-7 and collagen [16,17]. MMP-7 regulated collagen-I expression through Wnt/β-catenin or MAPK signaling in liver fibrosis [18] and kidney fibrosis [19]. Studies demonstrated that MMP-7 was crucial in the development of ILD and exacerbated fibrosis [20,21]. MMP-7 and collagen-I were also found to be colocalized in human lung fibrocytes [22]. MMP-7 exerts a significant role in the development of pulmonary fibrosis, including inflammation occurrence, extracellular matrix degradation, abnormal matrix repair, epithelial–mesenchymal transition, as well as tissue remodeling.

Recent clinical evidence indicated that MMP-7 levels were markedly elevated in RA-ILD patients’ serum, and began to increase at an early stage of the disease [23]. Compared with RA patients without ILD, the serum concentration of MMP-7 in RA-ILD patients was high [23]. However, it was unclear whether MMP-7 mediates ILD in RA, and whether MMP-7 can be used as a therapeutic target for RA-ILD. This study aims to explore the role of MMP-7 in the occurrence of ILD.

## 2. Materials and Methods

### 2.1. Bioinformatics Analysis

#### 2.1.1. Micro-Matrix Data

RA-ILD-associated mRNA expression datasets were screened in the Gene Expression Omnibus (GEO) database: firstly, use the website (https://www.ncbi.nlm.nih.gov/geo/, accessed on 6 April 2021); the keywords “rheumatoid arthritis interstitial lung disease”, “rheumatoiarthtitis” and “interstitial lung disease” were searched, and the conditions for further screening were “Homo sapiens”, “lung tissue”, “Expression profiling by array” and “Expression profiling by array”. The GSE21369 dataset was retrieved from the GEO database and included gene expression data from six normal lung tissue samples as well as twenty-three RA-ILD patients’ lung tissue samples.

#### 2.1.2. Identification of Differentially Expressed Genes (DEGs)

The GEO2R online platform (https://www.ncbi.nlm.nih.gov/geo/geo2r/, accessed on 29 March 2021) conducted a GEO2R analysis on the GSE21369 dataset, dividing the normal lung tissue and RA-ILD lung tissue into two groups. The GEO2R platform was utilized to identify differentially expressed genes (DEGs) between RA-ILD and normal lung tissues, and the results were downloaded. Subsequently, differential expression analysis of genes was performed using R language, and genes exhibiting significant differential expression between RA-ILD and normal lung tissues were identified. The screening criteria for DEGs were established as a significance level of *p* < 0.05 and |log FC| ≥ 1. Subsequently, heat maps and volcano plots were generated. DEGs with |log FC| ≥ 1 were designated as significantly upregulated, while those with |log FC| ≤ −1 were designated as significantly downregulated.

#### 2.1.3. Construction of the PPI Network

Proteins plays a crucial role in diverse physiological process, including material metabolism, energy metabolism and cell signal transduction through their mutual interactions. The STRING database (http://string-db.org) was utilized to construct a protein–protein interaction (PPI) network for the differentially expressed genes (DEGs) identified in RA-ILD lung tissues compared with normal lung tissues.

#### 2.1.4. Gene Ontology (GO) Analysis

Gene Ontology (GO) analysis provides explanations for the biological functions of certain genes from various aspects. Selecting differentially expressed genes (DEGs) in RA-ILD lung tissues compared with normal lung tissues, GO enrichment analysis was conducted on these DEGs using R language to explore the main functions and pathways that the significantly differentially expressed genes are involved in.

### 2.2. Materials and Reagents

Complete Freund’s Adjuvant (CFA) and IL-1β and IL-6 were obtained from Sigma-Aldrich Corp. (St. Louis, MO, USA). MMP-7 antibody (Cat: AM3358) was obtained from Abzoom Company (Wuppertal, Bergisches Land, Germany). Collagen-I antibody was purchased from proteintech (Rosemont, IL, USA). MMP inhibitor II (Cat: 444247) was purchased from Calbiochem (San Diego, CA, USA). TNF-α (Cat: 300-01A) was obtained from Peprotech (Cranbury, NJ, USA).

### 2.3. Culturing and Treating Human PMCs

The Met-5A (human PMC line) was obtained from the ATCC. These cells were maintained in an environment of 5% CO_2_ and 95% air at 37 °C, supplemented with 20% fetal calf serum in RPMI-1640 medium (Hyclone, Logan, UT, USA).

### 2.4. Cell Proliferation Testing

The CCK-8 kit (Dojindo, Kumamoto, Japan) was utilized to monitor cell proliferation. Cells were inoculated in a 96-well plate with 5000 cells per well, followed by the indicated treatment for 24 h. Subsequently, each well received 10 μL CCK-8 solution followed by incubation. Finally, 450 nm absorbance was recorded using a Multi-Mode micro-plate reader (BioTek, Winooski, VT, USA).

### 2.5. Patients’ Serum Samples’ Collection

The patients’ age range was 18~70 years old, and gender was not limited. Inclusion and exclusion criteria for RA-ILD were as follows: (1) Patients diagnosed with RA-associated interstitial pulmonary disease; (2) excluding other pulmonary diseases (such as COPD, asthma and other respiratory system diseases) and other connective tissue diseases. Inclusion and exclusion criteria for RA-no-ILD were as follows: (1) Patients diagnosed with rheumatoid arthritis without any pulmonary abnormalities seen on CT; (2) excluding other connective tissue diseases. After collecting fasting blood samples from patients, they were spun at 2000 rpm for 20 min. Serum, which is the supernatant, was separated from the blood cells at the bottom. The serum was then portioned into aliquots and preserved for subsequent studies.

### 2.6. siRNA and Lentivirus Plasmid Transfection

Following the manufacturer’s protocols, cells were transiently transfected with MMP-7 siRNA (IBSBIO, Shanghai, China) with the help of Lipofectamine RNAiMAX Reagent (Invitrogen, Carlsbad, CA, USA). The sequences of MMP-7 siRNA nucleotides were: sense (5′-3′): CGAUUAGUGUCAAAGGCUUTT; antisense (5′-3′): AAGCCUUUGACACUAAUCGTT. The expression of MMP-7 was detected by harvesting cell lysates 72 h after transfection. The MMP-7 siRNA was used for cell experiments.

For stable transfection, we constructed the MMP-7 shRNA lentivirus plasmid (GENECHEM, Shanghai, China), which was used for animal experiments. The target sequence of MMP-7 shRNA is 5′-TTGCAGGCATCCAGAAGTTAT-3′. The final dose of lentivirus is 5 × 10^7^ Tu/mL, and 400 μL MMP-7 or NC shRNA lentivirus were intrapleurally injected at days 11, 18, and 21 after the first CFA injection.

### 2.7. RA-ILD Animal Model

Twenty male Wistar rats (6–8 weeks) maintained in an air-conditioned room were randomly allocated into experimental and control groups. On day 0, experimental group rats received an injection of 0.1 mL CFA into their right hand paw [24,25], whereas control rats received an equivalent volume of PBS, followed by an intrapleural injection with MMP inhibitor II or MMP shRNA lentivirus at days 11, 18 and 21. For MMP inhibitor II, the rats’ weight was in the range 200 ± 10 g; for MMP-7 shRNA lentivirus, the rats’ weight was in the range 170 ± 10 g. The rats were euthanized with 1% Pentobarbital Sodium 28 days after the injection. Two-thirds of the left lung and one-half of the right middle lung were immersed in 4% formalin for fixation and subsequent embedding procedures. One-third of the left lung and one-half of the middle right lung were removed and stored at −80 °C until analysis by Western blotting. Measurements of paw swelling as well as body weight were taken prior to arthritis induction and every 2 to 4 days after the CFA injection until the experiment concluded. The thickness of the rat’s right hind paw was used to evaluate paw swelling.

### 2.8. Histological Analysis

To analyze morphology and assess collagen deposition, a hydroxyproline assay, Sirius red staining and Masson staining were conducted as previously outlined [26]. Following a 48 h fixation in 4% paraformaldehyde, the specific rat lung tissue samples were sliced midsagittal and embedded in paraffin. The slides were observed through a microscope (Carl Zeiss, Jena, Germany), and the images were processed using Image-Pro Plus 6.0 software.

### 2.9. Pulmonary Fibrosis Degree Score

According to Szapiel’s grading criteria [27], the Masson staining was evaluated to classify pulmonary fibrosis into 4 grades. Grade 0: the pathological sections showed normal lung tissue, indicating no fibrosis. Grade 1: a small amount of blue collagen was observed in the sections, and the lesion area was less than 20% of the total lung area, indicating mild interstitial pulmonary fibrosis. Grade 2: more collagen fibers and fibroblasts were observed in the pathological sections, and the alveolar structure was disordered, with the lesion area accounting for 20–50% of the total lung area, indicating moderate interstitial pulmonary fibrosis. Grade 3: multiple alveoli were fused in the sections, the lung parenchyma structure was disordered, a large amount of blue collagen was present, and the lung structure was destroyed, with the lesion area greater than 50%, indicating severe interstitial pulmonary fibrosis.

### 2.10. Western Blot

Following collection of PMCs or lung tissues, lysate samples were denatured and electrophoresed using 10% SDS-PAGE gels. Membranes were treated with antibodies targeting GAPDH (1:20,000), α-tubulin (1:10,000), collagen-I (1:2000), and MMP-7 (1:800), after being blocked with 5% skimmed milk (Sigma, St. Louis, MO, USA). Then HRP-conjugated secondary antibodies (1:2000) were incubated, followed by signal detection using ChemiDoc MP Image system (Bio-Rad Laboratories, Hercules, CA, USA). Bands were analyzed with Image-Pro Plus 6.0 software.

### 2.11. Statistical Analysis

To perform data analyses, GraphPad Prism 8.0 software was utilized. The results were presented as the mean ± SEM individually. Differences between the two sets of data were analyzed utilizing the *t*-test. Repeated measures one-way ANOVA was employed to compare the data of each group at various time points, with post hoc analysis conducted using the Bonferroni test.

## 3. Results

### 3.1. MMP-7 Was Involved in Formation of Extracellular Matrix Within Lung Tissue of RA-ILD

**Figure 1:**
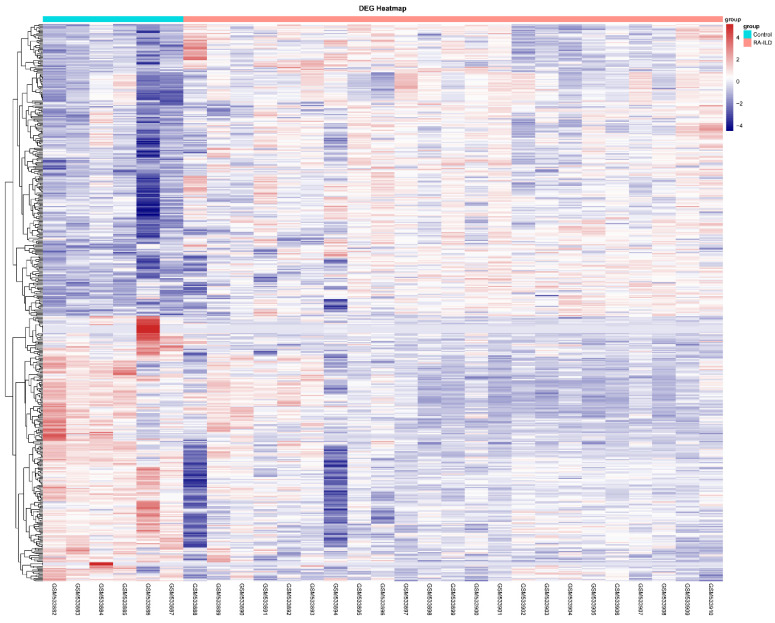
DEGs’ mRNA heatmap of RA-ILD lung tissue compared with normal lung tissue. Using the 29 samples in the GEO database, including 23 RA-ILD lung tissue samples and 6 normal lung tissue samples, a sample *t*-test analysis was conducted to detect the differentially expressed genes in RA-ILD lung tissue compared to normal lung tissue samples. The results were visualized in the form of heat maps and volcano plots. The R (V4.1) language analysis shows the expression status of mRNA in RA-ILD lung tissue and normal lung tissue. Blue represents low differential expression, and red represents high differential expression. *p* < 0.05, |log FC| ≥ 1.

**Figure 2:**
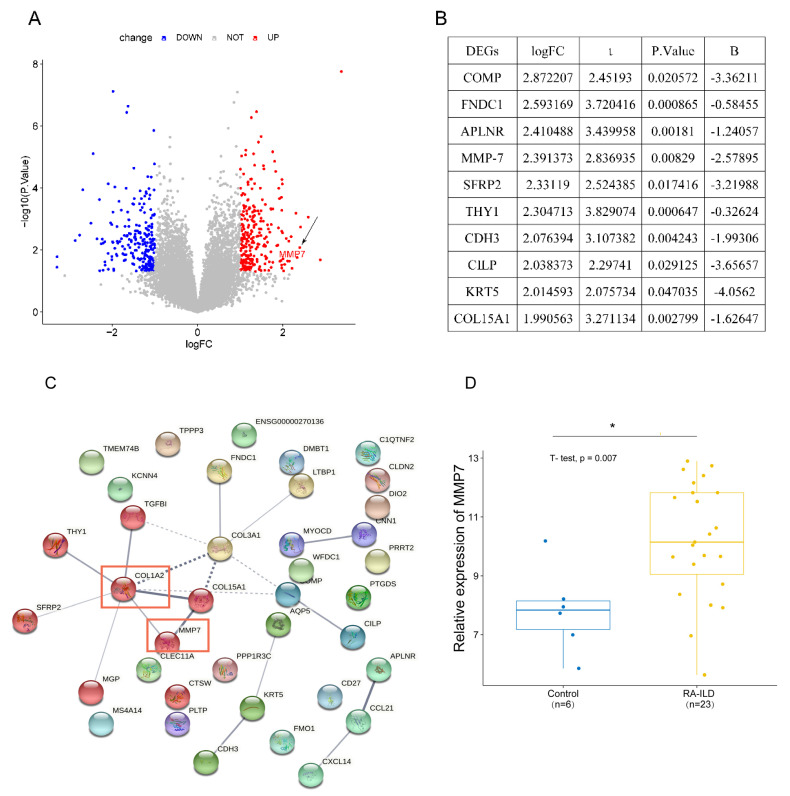
MMP-7 expression was significantly upregulated in RA-ILD lung tissues, and MMP-7 may interact with collagen-I. (**A**) Volcano plot shows differentially expressed genes (DEGs) in RA-ILD lung tissue compared to normal lung tissue. Red: DEGs that were significantly upregulated. Blue: DEGs that were significantly downregulated. *p* < 0.05, |log FC| ≥ 1. The black arrow indicates the MMP-7 gene. (**B**) The top 10 differentially expressed genes (DEGs) that were upregulated in RA-ILD lung tissues, namely COMP, FNDC1, APLNR, MMP-7, SFRP2, THY1, CDH3, CILP, KRT5 and COL15A1. (**C**) The interaction between MMP-7 and collagen-I within lung tissues of RA-ILD patients. PPI network map constructed based on log FC values > 1.5 of 38 DEGs in RA-ILD lung tissue samples. Solid lines represent strong interactions between proteins; dashed lines represent weak interactions between proteins. The red boxes respectively represent collagen-I and MMP-7. (**D**) MMP-7 showed high expression levels in the RA-ILD patients’ lung tissues. Control: normal lung tissue; RA-ILD: RA-ILD lung tissue; * *p* < 0.05.

**Figure 3:**
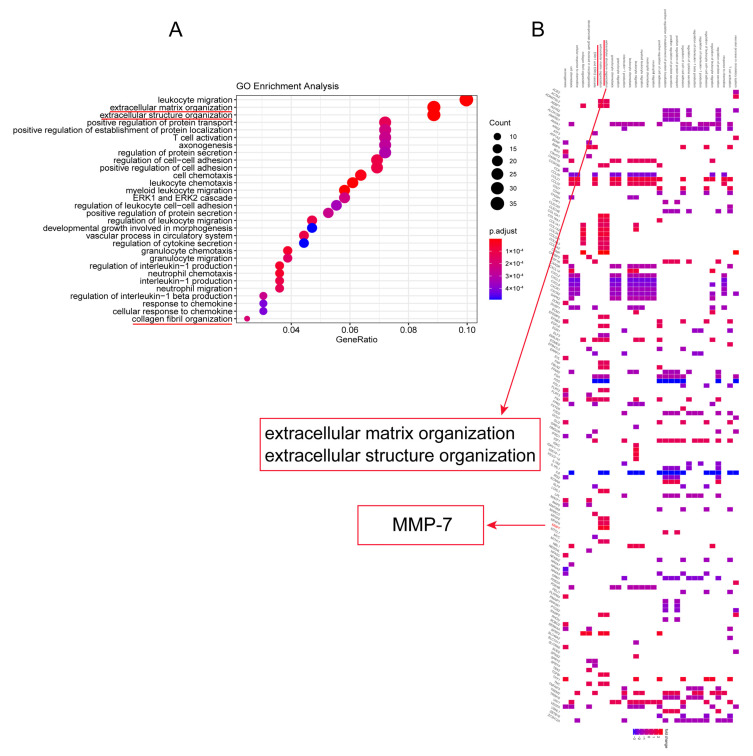
In RA-ILD lung tissues, MMP-7 was involved in the formation of the extracellular matrix. GO analysis was used to analyze the biological processes enriched by these DEGs. (**A**) GO analysis of RA-ILD lung tissue mRNA revealed their involvement in extracellular matrix and extracellular structure composition, ranking among the top three (marked with red underline). (**B**) MMP-7 was involved in the formation of the extracellular matrix and structure. Blue part indicates genes which were expressed at a lower level in RA-ILD compared to normal lung tissue samples, while the red part indicates the genes which were expressed at a higher level in RA-ILD compared to normal lung tissue samples.

To understand differentially expressed genes (DEGs) between RA-ILD lung tissues and normal lung tissues, bioinformatics analysis was performed on 29 samples including 23 RA-ILD lung tissue samples and 6 normal lung tissue samples in GEO database. Results were visualized as a heat map (Figure 1) and a volcano map (Figure 2A). The analysis showed that there were 496 DEGs in RA-ILD samples compared with normal samples, including 260 upregulated and 236 downregulated DEGs (Figure 1 and Figure 2A). The top 10 upregulated DEGs in RA-ILD lung tissue were sequenced, which were COMP, FNDC1, APLNR, MMP-7, SFRP2, THY1, CDH3, CILP, KRT5 and COL15A1 (Figure 2B). In total, 38 upregulated DEGs selected by the SRING website were used to construct the PPI network, and it was found that MMP-7 might interact with collagen-I in RA-ILD lung tissue (Figure 2C). A *t*-test was performed on lung tissue samples from 23 cases of RA-ILD and 6 cases of normal lung tissue, and MMP-7 was found to be highly expressed in lung tissue of RA-ILD (Figure 2D). Pathway enrichment analysis showed that these DEGs had great influence on white blood cell migration, extracellular matrix formation and extracellular structure composition, accounting for the top three (Figure 3A). MMP-7 was mainly involved in pathway-related composition of the extracellular matrix and structure (Figure 3B). Thus, the bioinformatics analysis indicated that MMP-7 was related with a high level of extracellular matrix especial collagen-I in RA-ILD lung tissues.

### 3.2. The RA-ILD-Related Inflammatory Factors Mediated Production of MMP-7 and Collagen-I in PMCs

**Figure 4:**
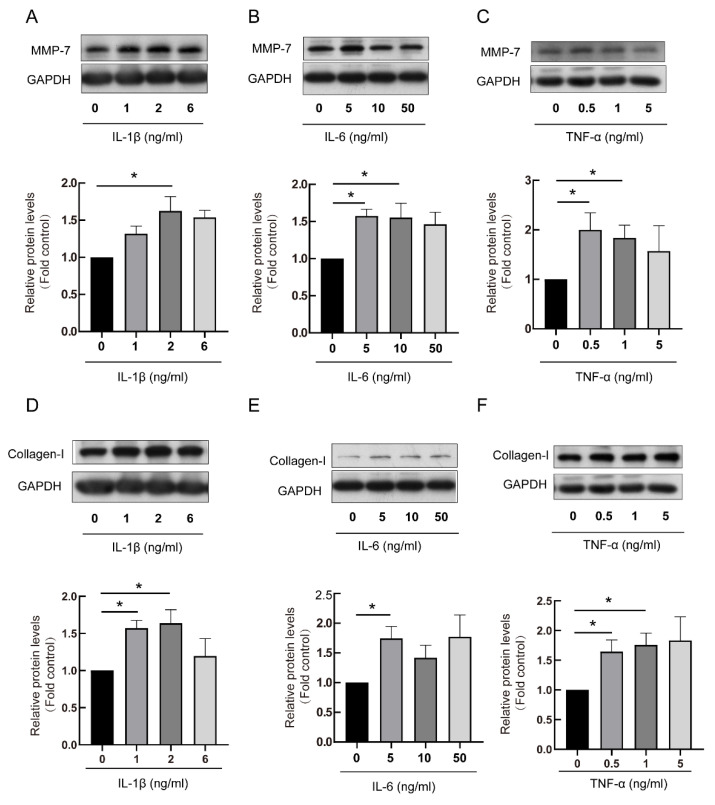
The RA-ILD-related inflammatory factors induced MMP-7 and collagen-I expression within PMCs. Identified inflammatory cytokines, including IL-1β, IL-6 and TNF-α, were used to treat cells. (**A**,**D**) PMCs were treated with IL-1β using different contents: 0, 1, 2 and 6 ng/mL for 48 h (n = 8). (**B**,**E**) PMCs were treated with IL-6 using different contents: 0, 5, 10 and 50 ng/mL for 72 h (n = 5). (**C**,**F**) PMCs were treated with TNF-α using different contents 0, 0.5, 1 and 5 ng/mL for 48 h (n = 3). MMP-7 (**A**–**C**) and collagen-I (**D**–**F**) protein contents were measured by Western blot. Data are expressed as mean ± SEM. * *p* < 0.05 vs. 0 ng/mL.

As RA-ILD-related inflammatory factors, IL-1β, IL-6 and TNF-α concentration increased in RA-ILD patients [21]. To investigate the relationships between these RA-ILD-related inflammatory factors and MMP-7, PMCs were stimulated by IL-1β, IL-6 and TNF-α, respectively. MMP-7 and collagen-I protein levels were assessed through Western blot. Results indicated that IL-1β, IL-6 and TNF-α promoted elevated expression of MMP-7 and collagen-I within PMCs (Figure 4).

### 3.3. RA-ILD Patients’ Serum Induced MMP-7 and Collagen-I Overexpression and Promoted Cell Proliferation in PMCs

**Figure 5:**
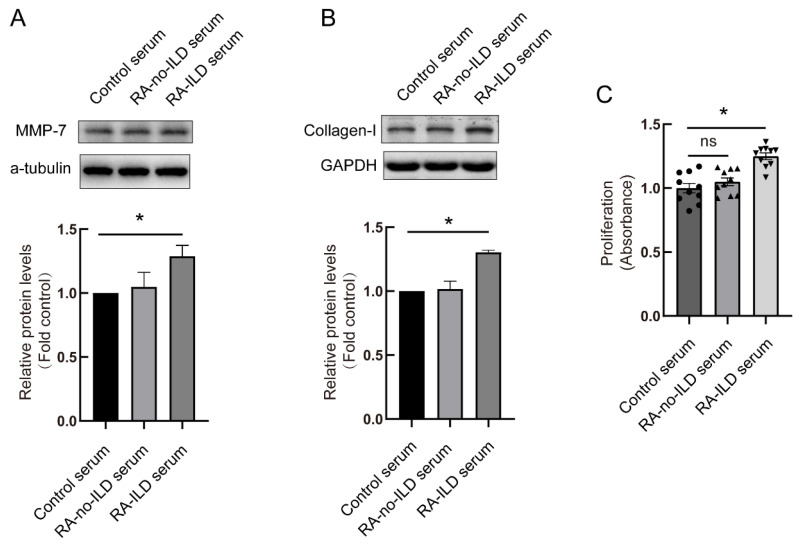
RA-ILD patients’ serum promoted production of MMP-7 and collagen by PMCs and cell proliferation. Control serum: the healthy volunteers’ serum treatment group. RA-no-ILD serum: RA patients’ serum treatment group. RA-ILD serum: RA-ILD patients’ serum treatment group. PMCs were incubated with 5% control serum, RA serum and RA-ILD serum for 48 h, MMP (**A**) and collagen-I (**B**) protein contents were measured by Western blot (n = 4). (**C**) PMCs were treated with 5% patients’ serum for 24 h, and then cell proliferation was detected by CCK-8 assay (n = 8). Data are expressed as mean ± SEM. * *p* < 0.05 vs. control serum.

To investigate MMP-7 function within RA-ILD development, PMCs were exposed to serum obtained from RA-ILD patients. The control serum was from healthy volunteers. Cells were treated by serum for 48 h, and MMP-7 and collagen-I protein levels in the cells were detected using Western blot. The serum of RA-ILD patients induced high level protein expressions of MMP-7 and collagen-I compared with healthy people (Figure 5A,B). Cell proliferation was also detected after serum treatment. As shown in Figure 5C, comparing the healthy volunteers’ serum treatment group and the RA-no-ILD patients’ serum treatment group, the RA-ILD patients’ serum treatment promoted PMC proliferation (Figure 5C). These data suggested that RA-ILD patients’ serum mediated MMP-7 and collagen-I overexpression and cell proliferation.

### 3.4. Knock-Down of MMP-7 Prevented Collagen-I Synthesis Induced by RA-ILD Patients’ Serum in PMCs

**Figure 6:**
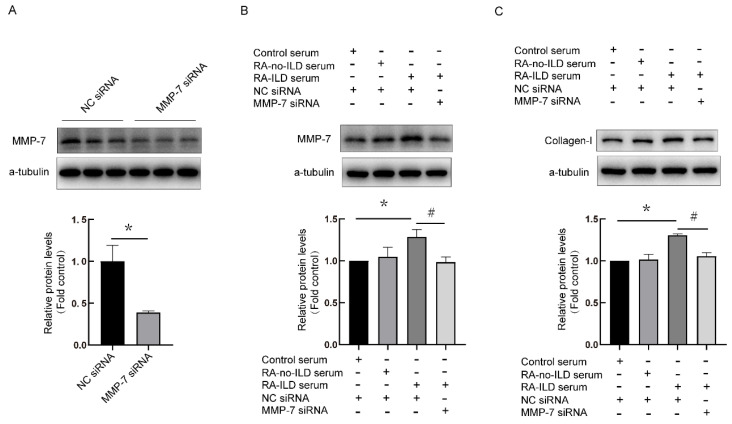
MMP-7 siRNA prevented MMP-7 and collagen-1 overexpression induced by RA-ILD patients’ serum in PMCs. (**A**) NC siRNA and MMP-7 siRNA were transfected into PMCs for 72 h (n = 6). MMP-7 protein expression was detected through Western blotting, which reflected the interference efficiency. (**B**,**C**) Following transfection with MMP-7 or NC siRNA, PMCs were exposed to control serum, RA-no-ILD serum and RA-ILD serum for 48 h, after which protein levels of MMP-7 (**B**) as well as collagen-I (**C**) were measured through Western blot. Results are shown as mean ± SEM (n = 4). * *p* < 0.05 vs. control serum; ^#^ *p* < 0.05 vs. RA-ILD serum.

Next, the relationship between MMP-7 and collagen-I in PMCs was investigated. MMP-7 siRNA was designed, and the interference efficiency was more than 50% (Figure 6A). Compared with the control, MMP-7 and collagen-1 expression were upregulated in the RA-ILD serum treatment group. However, expressions of collagen-I protein were reduced in the RA-ILD group by MMP-7 siRNA pretreatment (Figure 6B,C). These results suggested that MMP-7 mediated collagen-I synthesis in human PMCs treated by serum from RA-ILD patients.

### 3.5. CFA Induced the Development of Arthritis In Vivo

**Figure 7:**
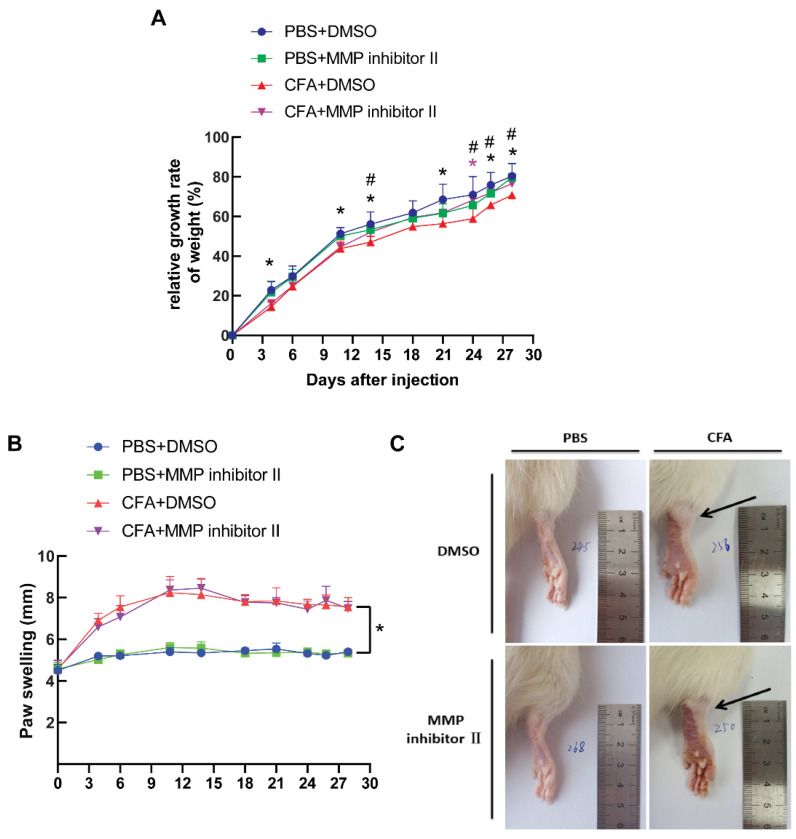
The CFA-induced joint arthritis. Male Wistar rat was injected with 0.1 mL CFA or PBS to the right hind paw at day 0, then intrapleural injections with MMP inhibitor II (0.7 mg/kg) or DMSO at days 11,18 and 21. Paw swelling was measured on day 28 after the first CFA injection by using a Vernier caliper to assess the thickness of each rat’s right hind paw. Rat weight (**A**) and paw swelling (**B**) were measured every 2–4 days. (**C**) The gross view of rat’s right hind paw, with the swollen ankle joint indicated by the black arrow. n = 5. * *p* < 0.05 for CFA + DMSO vs. PBS + DMSO; ^#^ *p* < 0.05 for CFA + MMP inhibitor II vs. CFA + DMSO.

To study the relevance of cellular findings in the disease, animal models were made. Rats were injected with CFA as described in the Materials and Methods section, and the weight of the rats was measured every 2–4 days. Compared with the control group, the weight gain ratio of the CFA model rats was relatively low, but this was prevented by using MMP-7 inhibitor II in the rats. At day 28 after CFA injection, the weight gain ratio was similar to that of the control group (Figure 7A).

One day after CFA injection, inflammatory reactions were observed in rats’ hind feet and joints. In comparison to the normal control group, CFA-treated rats exhibited obvious redness and swelling of the hind foot accompanied with ankle joint enlargement. At days 3–4, the CFA-treated rats showed redness and inflammation of the foot paw, movement disturbance and licking of the affected foot paw, and the foot thickness of the rats increased significantly. At the end of the study, the foot thickness of rats was still markedly elevated in the CFA-treated rats compared with the normal control group (Figure 7B,C). These data indicated that CFA led to the development of arthritis.

### 3.6. MMP-7 Inhibitor Attenuated CFA-Induced Subpleural Lung Fibrosis In Vivo

**Figure 8:**
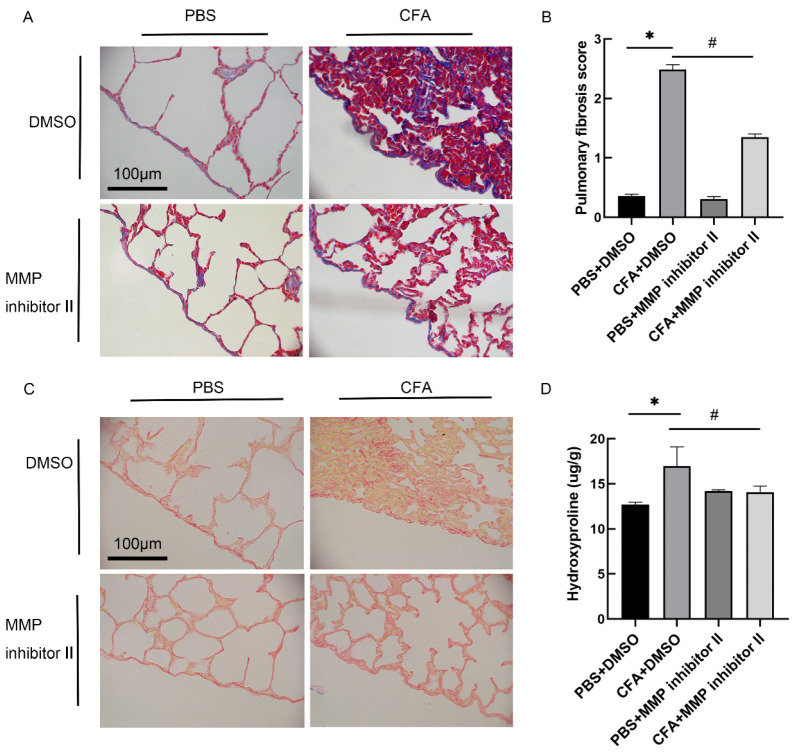
MMP-7 inhibitor attenuated CFA-induced subpleural fibrosis in vivo. Wistar rat RA-ILD model was established by injecting 0.1 mL of CFA or PBS into the right hind paw on day 0, followed by intrapleural administrations of MMP inhibitor II or DMSO on days 11, 18 and 21. Rats were euthanized at days 28 after the first CFA injection, and lung tissues were harvested for further examination. (**A**) Representative images of Masson staining in subpleural pulmonary tissues from control mice as well as CFA-injected mice treated with MMP inhibitor II or DMSO. (**B**) Changes in pulmonary fibrosis severity score for lung sections. (**C**) Typical images of Sirius red stain in subpleural lung tissues from control mice and CFA-injected mice treated with MMP inhibitor II or DMSO. The collagen fibers were visualized by polarized light microscopy. (**D**) Changes in hydroxyproline level within lung tissue. Results are shown as mean ± SEM (n = 5). * *p* < 0.05 vs. PBS + DMSO, ^#^ *p* < 0.05 vs. CFA + DMSO.

To investigate the effect of MMP-7 inhibitor in CFA-induced rat subpleural fibrosis, an RA-ILD animal model was treated with MMP inhibitor II intrapleural injection as described in the Materials and Methods section. Lung tissue sections were stained using Masson and Sirius scarlet staining. The pulmonary fibrosis degree score and concentration of hydroxyproline in lung tissue were also measured. Compared with the normal control group, collagen deposition and alveolar structure rupture were observed in the subpleural and alveolar interstitial of the CFA-treated group. MMP-7 inhibitor intervention reduced collagen deposition and structural damage (Figure 8A), and MMP-7 inhibitor attenuated the CFA-induced pulmonary fibrosis score (Figure 8B). The same results were also found in lung tissue Sirius scarlet staining and hydroxyproline content (Figure 8C,D).

### 3.7. MMP-7 shRNA Alleviated CFA-Induced Subpleural Lung Fibrosis

**Figure 9:**
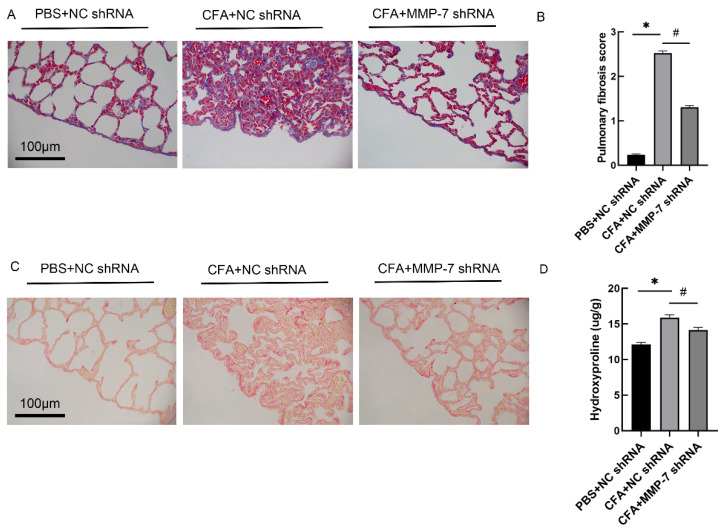
MMP-7 shRNA lentivirus attenuated CFA-induced subpleural fibrosis in vivo. Wistar rat RA-ILD model was induced by injection of 0.1 mL CFA or PBS administered to the right rear paw at day 0, then intrapleural injections with MMP shRNA or NC shRNA lentivirus at days 11, 18 and 21. Rats were euthanized at days 28 after the first CFA injection, lung tissues were harvested for histological examination. (**A**) Representative images of Masson staining in subpleural pulmonary tissues from control mice and CFA-injected mice treated with MMP shRNA or NC shRNA lentivirus. (**B**) Changes in pulmonary fibrosis severity score for lung sections. (**C**) Typical images of Sirius red stain in subpleural lung tissues from control mice and CFA-injected mice treated with MMP shRNA or NC shRNA. The collagen fibers were visualized by polarized light microscopy. (**D**) Changes in hydroxyproline in lung tissue reflect the content of collagen. n = 5. * *p* < 0.05 vs. PBS + NC shRNA, ^#^ *p* < 0.05 vs. CFA + MMP-7 shRNA.

To further explore the role of MMP-7, lentivirus vectors carrying MMP-7 shRNA were constructed. The MMP-7 expression was reduced after MMP-7 shRNA lentivirus intrapleural injection (Figure S1). In the CFA-induced RA-ILD model rats, MMP-7 shRNA lentivirus was injected into the pleural cavity, and Masson staining as well as Sirius scarlet staining were performed. The pulmonary fibrosis score and hydroxyproline content were measured as described in the methods. As shown in Figure 9, MMP-7 shRNA alleviated CFA-induced lung inflammation and pulmonary fibrosis in rats.

## 4. Discussion

In this study, bioinformatics analysis revealed that MMP-7 expression was significant elevated in the lung tissue of RA-ILD, and MMP-7 may potentially interact with collagen-I. Next, in vitro experiments revealed that RA-related cytokines IL-1β, IL-6 and TNF-α promoted MMP-7 and collagen-I expression in PMCs. Serum obtained from patients with RA-ILD also induced upregulation of MMP-7 and collagen-I in PMCs. Moreover, suppressing MMP-7 using MMP-7 siRNA or MMP inhibitor prevented collagen-I synthesis in PMCs. In vivo, CFA induced rats’ arthritis and lung inflammation, but MMP-7 inhibitor and MMP-7 shRNA attenuated CFA-induced lung inflammation and fibrosis.

MMPs play significant roles in cell migration, invasiveness and angiogenesis. MMPs also take part in epithelial–mesenchymal transition, the TGF-β signaling pathway and extracellular matrix production. MMP-7 is a secreted protein that depends on zinc and calcium [28]. It is comprised of a catalytic domain and a pro-domain, and exists in an inactive enzyme precursor form. Its activation requires the hydrolysis of the 9 KDa N-terminal propeptide region to form a 19 KDa active form. MMP-7 can hydrolyze various extracellular matrices, including casein, gelatin, fibronectin and proteoglycans, and can also hydrolyze non-extracellular matrices such as E-cadherin and apoptotic ligand (FasL). MMP-7 is typically considered as a downstream target within the Wnt/β-catenin signal pathway, which regulates its expression through β-catenin and thereby regulates the formation of fibrosis [29]. Previous studies reported that β-catenin/MMP-7 regulated collagen-I expression via the EMT process in liver fibrosis [18]. MMP-7 overexpression promotes collagen-1 synthesis via the MAPK signaling pathway in kidney fibrosis [19]. Moreover, the effects of inflammatory factors, including IL-1β, IL-6 and TNF-α, promote the progression of RA and induce collagen-I synthesis via signaling pathways such as Wnt/β-catenin and JAK/STAT [24,29,30,31,32]. This knowledge and the literature built a foundation for the role of MMP-7 in RA-ILD.

As a chronic systemic autoimmune disease, RA is known for causing joint inflammation and extra-articular manifestations, including pulmonary disorders such as ILD. Histological characteristics of RA-ILD included subpleural inflammation and collagen deposition, accompanied by varying degrees of fibrotic change, which is generally irreversible. The expression of MMP-7 was elevated in both human idiopathic pulmonary fibrosis (IPF) and mouse fibrosis models. Moreover, when MMP-7 was knocked out in fibrotic mouse models, the fibrosis was attenuated [20,21]. MMP-7 not only promoted fibrosis in ILD, but also promoted fibrosis in other tissues, such as kidney fibrosis [33,34], liver fibrosis [35] and myocardial fibrosis [36]. The MMP-7 level was found to be increased in IPF patients’ serum and also positively correlated with the progression of IPF in previous clinical studies [37,38,39]. MMP-7 may have the potential to become a biomarker for RA-ILD.However, this should be validated through more prospective, large-sample and multicenter cohort studies [40,41]. In the present study, we demonstrated that MMP-7 mediated collagen-I synthesis and was involved in the occurrence of ILD; inhibition of MMP-7 prevented pulmonary fibrosis in RA-ILD models. Thus, we thought that MMP-7 may also serve as a promising therapeutic target for RA-ILD.

UIP as well as NSIP are pathological types of ILD [42]. In RA-ILD patients, the pathological types included 56% UIP and 33% NSIP [9]. The pathological type of ILD determines the prognosis and different therapies. UIP is less sensitive to the use of glucocorticoids with worse prognosis compared with NSIP. UIP is the most pathological type in RA-ILD, and the fibrosis is often located in the subpleural lung in patients. Previous cohort clinical studies revealed that IPF and RA-ILD patients showing a UIP pattern had high serum levels of MMP-7 compared with that in NSIP pattern patients [43]. Moreover, UIP patients exhibited a quickly reduced lung diffusing capacity for carbon monoxide when compared with NSIP patients [43]. In a certain sense, monitoring of MMP-7 may improve diagnostic accuracy for RA-ILD exhibiting a UIP pattern as well as be helpful for evaluation of the prognosis of patients. ECM remodeling is instrumental in UIP pathogenesis. MMP-7, as a kind of ECM-modulating enzyme, has the function of regulating collagen expression and contributes to the pathogenesis of RA-ILD.

For our animal models, a limitation is that the specific pathological type of ILD was not identified because the pathological changes in the models did not exhibit classical manifestations of NSIP or UIP. However, the prominent collagen fiber deposition and minimal inflammatory changes observed in the subpleural regions suggested that the pathological type in our animal models resembled UIP. Another limitation of our experiments was animal gender selection, which may lead to incomplete research outcomes or potential misinterpretations of the data. To address this issue, we plan to include both male and female animals in our future experiments.

In summary, this study uncovered that MMP-7 mediated collagen-I synthesis in PMCs, and the inhibition of MMP-7 attenuated subpleural fibrosis in experimental RA-ILD.

## Data Availability

Data will be available upon request.

## Supplementary Materials

The following supporting information can be downloaded at: https://www.mdpi.com/article/10.3390/biomedicines13071581/s1, Supplemental Methods and Figure S1: The MMP-7 expression was reduced after MMP-7 shRNA lentivirus intrapleural injection.

## Abbreviations

The following abbreviations are used in this manuscript:

CFA	Complete Freund’s adjuvant
DEGs	Screening of differentially expressed genes
MMP-7	Matrix metalloproteinase-7
PPI	Protein–protein interaction
PMCs	Pleural mesothelial cells
RA-ILD	Rheumatoid arthritis-related interstitial lung disease
RA	Rheumatoid arthritis
