# Supplementary material for: Inhibition of Matrix Metalloproteinase-7 Attenuates Subpleural Fibrosis in Rheumatoid Arthritis-Associated Interstitial Lung Disease
Source: Biomedicines. 2025 Jun 27;13(7):1581. doi: 10.3390/biomedicines13071581 (PMC12292077; doi:10.3390/biomedicines13071581)
Supplement: Supplementary file 1 [file biomedicines-13-01581-s001.zip › biomedicines-3648517-supplementary.pdf]

Supplementary data

Title: Inhibition of matrix metalloproteinase-7 attenuates subpleural fibrosis in rheumatoid arthritis-associated interstitial lung disease

Authors: Li Xiong [1], Li-Mei Liang [1,2], Shu-Yi Ye [1], Xiao-Lin Cui [3], Shi-He Hu [3], Chen-Yue Lian [1], Wen-Jia Sun [1], Yang-Ping Lv [3], He-De Zhang [3], Meng Wang [3], Fei Xiang [1,2], Liang Xiong [1,2], Hong Ye [2,3], Wan-Li Ma [1,2] and Lin-Jie Song [1,2*]

1 Department of Respiratory and Critical Care Medicine, Union Hospital, Tongji Medical College, Huazhong University of Science and Technology, Wuhan 430022, China

2 Key Laboratory of Respiratory Diseases of National Health Commission of China, Wuhan 430030, China

3 Department of Pathophysiology, School of Basic Medicine, Tongji Medical College, Huazhong University of Science and Technology, Wuhan 430030, China

* **Correspondence:** songlinjiewhs@hust.edu.cn

Contents:

Supplemental Methods:

1. Immunohistochemical staining

Supplemental Figures:

Figure S1: The MMP-7 expression was reduced after MMP-7 shRNA lentivirus intrapleural injection.

Supplemental Methods:

1. Immunohistochemical staining: Lung sections (5 μm) were exposed to anti-MMP-7 primary antibody (1:1000, Cat: AM3358, Abzoom Company) at 4 ℃ overnight after dewaxed and antigen retrieval. And then incubated with secondary antibodies. The DAB solution, hematoxylin and mounting medium was applied to the slides and the pictures were taken using digital camera microscope.

Supplemental Figures:

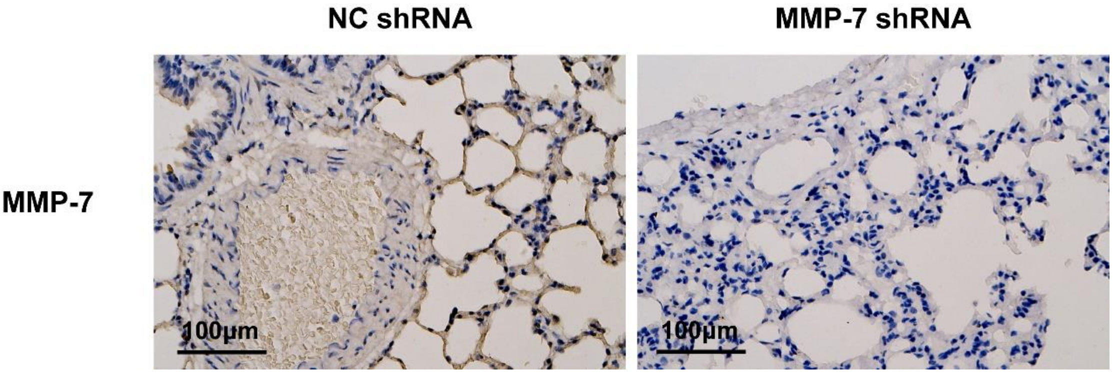

Supplementary Figure S1. The MMP-7 expression was reduced after MMP-7 shRNA lentivirus intrapleural injection. Wistar rats were intra-pleural injections with NC shRNA or MMP-7 shRNA lentivirus at days 11, 18 and 21. Rats were euthanized at days 28 and lung tissues were harvested. Lung sections were stained with immunohistochemical staining using antibody against MMP-7. Brown color shows MMP-7. Blue color shows nuclei. Original magnification, x400.
